# Granulomas Formation in Lymph Nodes, Liver and Spleen in Adult-Onset Still’s Disease: A Case Report

**DOI:** 10.4021/jocmr1281e

**Published:** 2013-02-25

**Authors:** Stelios F. Assimakopoulos, Vassilios Karamouzos, Christos Papakonstantinou, Vassiliki Zolota, Chryssoula Labropoulou-Karatza, Charalambos Gogos

**Affiliations:** a Department of Internal Medicine, University Hospital of Patras, Rion-Patras 26504, Greece; b Department of Pathology, University Hospital of Patras, Rion-Patras 26504, Greece

**Keywords:** Adult-onset Still’s disease, Granulomatous, Lymphadenitis, Hepatic granulomas, Anakinra

## Abstract

Tissue granulomas formation in adult-onset Still’s disease (AOSD) is extremely rare. We describe a case of AOSD associated with formation of granulomatous lesions in lymph nodes, liver and presumably spleen. The high dose steroid-dependent nature of our patient’s illness, characterized by disease relapses when methylprednisolone dose was reduced below 10 mg/d, was overwhelmed with institution of anakinra (100 mg/d). The histologic finding of granulomas formation in lymph nodes, liver or spleen should not deter the consideration of AOSD as a potential diagnosis in a compatible clinical context; however, other more common etiologies of tissue granulomas formation should be first excluded.

## Introduction

Adult-onset Still’s disease (AOSD) is a systemic inflammatory disorder clinically characterized by high daily fevers, arthralgia, skin rash, lymphadenopathy, and hepatosplenomegaly. Its diagnosis requires exclusion of any infection, malignancy, or other rheumatic disorder known to mimic AOSD in its clinical features [1]. Liver involvement expressed as liver enzymes elevations and/or hepatomegaly is found in over 60% up to 85% of patients with AOSD [2, 3], whilst lymphadenopathy and splenomegaly is found in about 65% of AOSD patients [1, 4, 5]. Despite its diagnosis is based upon clinical criteria, lymph node or liver biopsies could be performed to exclude other diagnostic considerations such as lymphomas or autoimmune hepatitis, respectively.

Lymphadenopathy in AOSD is histologically characterized by an intense, paracortical immunoblastic hyperplasia [1, 4, 5]. Hepatic biopsies of AOSD patients with liver dysfunction show periportal mononuclear infiltrates, Kupffer-cell hyperplasia, lobular inflammation, focal hepatocellular degeneration, periportal fibrosis and ground glass-like cytoplasmic inclusions, whilst massive or submassive hepatic necrosis has been also described. The formation of granulomas in involved organs in AOSD is an extremely rare pathological manifestation of this disease. We have recently described the first case of a necrotizing granulomatous lymphadenitis associated with AOSD [6], whereas, to the best of our knowledge, there are no previous reports of hepatic granulomas associated with AOSD.

We describe here a case of AOSD with lymphadenopathy, hepatomegaly and splenomegaly, which were histologically characterized by granulomas formation.

## Case Report

A 54-year-old female patient, with unremarkable past medical history except hypertension and depression, was admitted to our department because daily fever up to 39 °C associated with rigors and notice of a palpable painful mass in her right axilla. She reported no previous close animal contact or injury.

On admission, the patient’s vital signs were 38.9 °C, HR 92 bpm, RR 14 b/min BP 130/85 mmHg. Oxygen saturation breathing ambient air was 97%. Physical examination revealed a 5 cm painless mass in the right axilla, palpable liver 3 cm below the right costal margin and just palpable spleen below the left costal margin.

Initial laboratory evaluation showed WBC count 13.2 × 10^9^/L, with predominant neutrophils (82%), hematocrit 28.4% hemoglobin 9.2 g/dL (MCV = 80 and MCH = 26) and platelet count 587 × 10^9^/L. Prothrombin and partial thromboplastin times were normal and d-dimers were increased at 1.20 μg/mL. The blood biochemical test results including serum angiotensin converting enzyme were normal except of increased serum globulins at 3.7 g/dL. Protein electrophoresis was normal. Serum CRP was increased at 17.5 mg/dL, ESR at 130 mm/h, fibrinogen at 808 mg/dL and ferritin at 336 mg/dL. Thyroid function tests were normal. Urinalysis and 24-hour urinary calcium and protein excretion were unrevealing. Electrocardiogram, chest X-ray, echocardiogram and arterial blood gas were also normal. A detailed ophthalmologic examination including slit lamp eye examination, fundoscopy, Rose Bengal and Schirmer’s tests was unrevealing.

**Figure 1:**
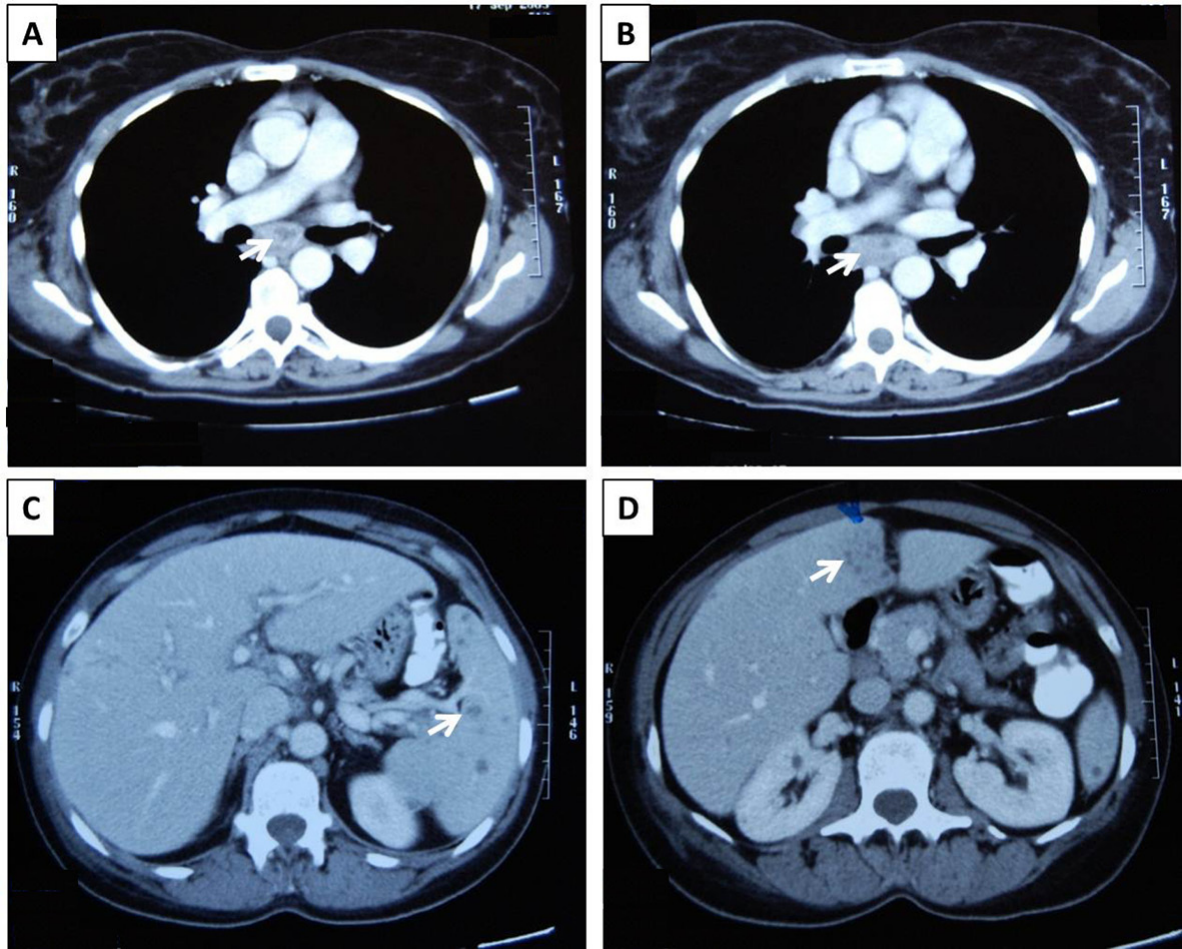
Thoracic and abdominal CT scans from the presented patient: Enlarged mediastinal lymph nodes with a hypodense center (A, B, white arrows), and multiple hypodense splenic (C, white arrow) and hepatic (D, white arrow) lesions were detected.

A full body CT scan revealed enlarged lymph nodes in the right axillary, mediastinal (Fig. 1A, B) and retroperitoneal region, most with a hypodense center, and hepatosplenomegaly with multiple hypodense splenic and hepatic lesions (Fig. 1C, D). There was no evidence of lung parenchymal disease, pleural or pericardial effusions or ascites, whilst the vascular perfusion of abdominal organs was normal. A dual phase hepatic CT scan showed a diffuse heterogeneity in hepatic arterial perfusion with multiple hypodense lesions not enhancing in arterial face. A Gallium-67 scan showed increased radiotracer uptake in right axillary, mediastinal and retroperitoneal lymph nodes and in the right hepatic lobe and the spleen. An ^18^F-FDG PET/CT scan showed multiple foci of increased metabolic activity in the lymph nodes of the right axillary and subcarinal regions, in the right hepatic lobe and the spleen. A performed digital mammography was unrevealing.

All sets of blood cultures (at least six), urine and stool cultures were negative. Antibodies for hepatitis A, B, C, coxsackie, echo, herpes simplex virus, Epstein-Barr, cytomegalovirus, human immunodeficiency virus, human T-lymphotropic virus-1 and -2, Yersinia enterocolitica Entamoeba histolytica, Bartonella henselae, Francisella tularensis Leishmania donovani, Coxiella burnetii and Rickettsia coronii were negative. Wright and RPR tests as well as the tuberculin skin test were also negative.

The patient underwent upper and lower gastrointestinal tract endoscopies, which detected no pathology. A bronchoscopy was also performed without pathologic findings, whilst examination of bronchoalveolar lavage with Gram and Ziehl-Neelsen stains, cultures, polymerase chain reaction for mycobacterium tuberculosis and cytology were unrevealing. The CD4:CD8 ratio in the bronchoalveolar lavage was normal.

A full immunologic screening with rheumatoid factor, antinuclear antibodies, antibodies to double-stranded DNA, anti-Sm, anti-Ro/SSA, anti-La/SSB, anti-RNP, anti-Jo-1, anti-Scl-70, anti-histones, anti-mitochondrial antibodies, anti-smooth muscle antibodies, cytoplasmic-antineutrophil cytoplasmic antibody (ANCA), perinuclear-ANCA, anti-transglutaminase, anti-cardiolipin, and lupus anticoagulant was negative. Serum complement and levels of immunoglobulins (Igs; IgA, IgG, IgM, IgE, IgD) were normal. The results of genetic testing for mutation of the familial Mediterranean fever gene (M694V, V726A, M694I, M680I, and E148Q) were negative. In addition, the results of a full tumor marker profile were also normal.

**Figure 2:**
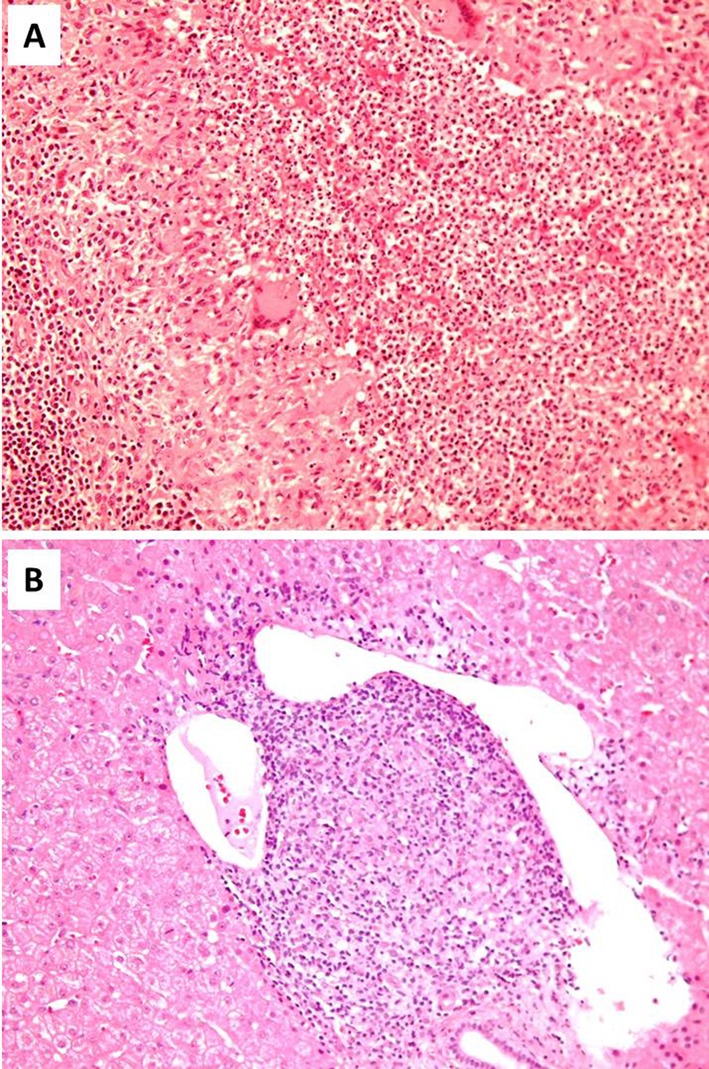
Lymph node and liver biopsies from the presented patient: Formation of histiocytic granuloma with central suppurative necrosis in lymph node (A) and histiocytic granuloma in liver (B) (H&E, A: × 200, B: × 200).

The patient underwent surgical excision of the enlarged right axillary lymph nodes for microbiological and histological examination. Histology revealed a granulomatous lymphadenitis with central suppurative necrosis (Fig. 2A). Gram, Giemsa, Ziehl-Neelsen, Grocott methenamine silver stains for detection of common bacteria, mycobacteria and fungi were all negative. Immunohistochemical studies for lymphoproliferative disease was unrevealing. In addition, lymph node was examined with PCR for mycobacterium tuberculosis, atypical mycobacteria (africanum I/II, microti, carnetti, bovis, avium complex), fungi and Bartonella hensellae, in addition to application of culture techniques, without detection of any pathogen. A CT-scan-guided liver biopsy was performed in hepatic hypodense lesions, which demonstrated the presence of hepatic histiocytic granulomas (Fig. 2B). A gastrocnemius muscle biopsy was also performed without identification of sarcoid granulomas.

The peripheral blood film showed no evidence of lymphoproliferative disorders. Bone marrow biopsy and immunophenotypic analysis revealed no pathologic findings. Peripheral blood immunophenotypic analysis revealed T-lymphopenia. Beta -2 microglobulin levels, serum and urine kappa and lambda light chains and urine Bence-Jones protein test were unrevealing.

The patient had been initially empirically treated with antimicrobial therapy consistent of ciprofloxacin and clindamycin without disease control. During hospitalization episodic disease flares characterized by SIRS and spikes of increased markers of inflammation were recorded, which were treated with step up of antimicrobial therapy to cover potential intra-hospital pathogens (linezolide - meropenem) and hydrocortisone 50 mg qid. After completion of the above described extensive diagnostic work-up which demonstrated no evidence of an infectious etiology of our patient’s symptoms, we decided to administer corticosteroids (32 mg methylprednisolone) as a therapeutic trial. The patient presented an immediate improvement after institution of this therapy becoming afebrile and with normalization of serum inflammatory markers. She entered a very slow corticosteroid tapering scheme (reduction of 2 mg of methylprednisolone every month) with close clinicolaboratory follow up and she presented gradual resolution of lymphadenopathy. However, the disease relapsed with reappearance of fever, lymphadenopathy and increased inflammatory markers, when methylprednisolone dose was reduced below 10 mg/d. Methylprednisolone dose was again increased to 16 mg/d with immediate response of fever and gradual resolution of lymphadenopathy, followed by slow tapering of corticosteroids. Unfortunately, a second relapse occurred again with methylprednisolone dose reduction below 10 mg/d. A trial of a non-steroidal anti-inflammatory agent (ibuprofen 800 mg daily) added in methylprednisolone treatment, which was again raised to 16 mg/d, failed to prevent a new disease flare with methylprednisolone dose reduction below 10 mg/d. After this third disease relapse, anakinra (100 mg of daily) was added concurrently with methylprednisolone dose escalation in 16 mg daily. The patient was treated with 16 mg/d of methylprednisolone for two months and entered a slow corticosteroid tapering scheme (reduction of 2 mg of prednisolone every month). She is currently treated with 6 mg/d of methylprednisolone and is free of symptoms with resolution of lymphadenopathy, and absence of radiotracer Gallium-67 up-take. The hypodense hepatic and splenic lesions are still discernible on contrast CT imaging; however, a repeated CT-guided biopsy of liver lesions detected no granulomas but only evidence of pericentral and pericellular fibrosis. Liver function tests are within normal limits.

## Discussion

We describe a previously healthy female patient presenting with prolonged fever, lymphadenopathy, hepatosplenomegaly, increased inflammatory markers and disease flares characterized by SIRS, immediate clinico-laboratory response to corticosteroids but requiring prolonged therapy and high corticosteroid dose (at least 10 mg/d of methylprednisolone) for disease control. On CT scan imaging enlarged axillary, mediastinal and retroperitoneal lymph nodes with a hypodense center and multiple hypodense hepatic and splenic lesions were detected. Lymph node and liver biopsies revealed the formation of granulomatous lesions in these organs. Despite spleen lesions were not biopsied their imaging characteristics were similar to the hepatic lesions and it would be reasonable to assume that their histologic characteristics would be also the same. Interestingly, lymph node granulomas were characterized by central suppurative necrosis.

Several diseases of benign or malignant etiology can present as necrotizing granulomatous adenitis [7-9]. Benign causes include infectious diseases (bacterial, viral, fungal or parasitic), autoimmune disorders like systemic lupus erythematosus, autoinflammatory diseases, and idiopathic causes like Kikuchi’s disease and sarcoidosis. Malignant disorders, mainly lymphoid malignancies (Hodgkin and non-Hodgkin disease) and rarely metastatic carcinomas should always be included in the differential diagnosis and thoroughly investigated. The presence of variable degrees of suppuration although being suggestive of specific diagnoses like Yersinia pseudotuberculosis infection or tuberculosis does not preclude other potential causes of necrotizing granulomatous adenitis [7].

In the described patient, no infectious etiology could be detected by serological, microbiological, histological and molecular methods. Lymphoid malignancies, either non-Hodgkin or Hodgkin disease, were excluded based on lymph node histologic and immunohistochemical studies, bone marrow aspiration, biopsy and immunophenotypic analysis, and peripheral blood smear examination and immunophenotypic analysis.

Sarcoidosis was an important diagnostic consideration in the presented patient. The typical histological feature of sarcoidosis is the formation of non-necrotizing granulomata [10]; however, the existence of necrotizing sarcoid granulomatosis has also been described since 1973 [11]. Normal serum angiotensin converting enzyme (ACE) levels, absence of hypercalciuria, normal pulmonary function tests, slit lamp eye examination, CD4 to CD8 ratio in BAL, and no detection of sarcoid granulomata in gastrocnemius muscle biopsy [12], made this diagnosis unlikely.

Regarding other potential diagnoses, systemic lupus erythematosus was excluded by the absence of autoantibodies, Wegener’s disease and Churg-Strauss syndrome were ruled out based mainly on clinical criteria combined with negative ANCAs [13]. Kikuchi-Fujimoto disease (KFD), was excluded based on the prolonged and relapsing clinical course of our patient and the histological features of affected lymph nodes with significant infiltration by neutrophils [14]. Amongst autoinflammatory diseases, the FMF was excluded by appropriate genetic testing, the hyper-IgD syndrome by normal serum IgD and IgA lavels and the periodic fever with aphthous stomatitis, pharyngitis, and adenitis syndrome by clinical criteria [15-17].

After the aforementioned extensive diagnostic work-up and rule out of all potential alternative diagnostic considerations, AOSD, an inflammatory disorder that could be expressed with daily fevers, increased inflammatory markers, lymphadenopathy and hepatosplenomegaly, in the absence of positive autoantibodies, came into play as an important diagnostic consideration. Disease presentation in the patient was not typical for AOSD because it lacked common features such as arthralgias, skin rash, sore throat and significant serum hyperferritinemia [14]. However, diagnosis of AOSD was based on fulfillment of the commonly used high sensitive Yamaguchi criteria (93.5% sensitivity) and exclusion of any other diagnostic consideration including any infectious, malignant, or rheumatic disorder known to mimic AOSD in its clinical features (Table 1) [1, 15]. The high dose steroid-dependent nature of the presented patient’s illness was finally overwhelmed with institution of anakinra, an interleukin-1 receptor antagonist, which has been previously shown to be an effective, safe and steroid sparing treatment option for AOSD [18].

**Table 1:** Accordance of the Presented Patient With the Yamaguchi Criteria for the Diagnosis of Adult-Onset Still’s Disease

Yamaguchi criteria (require the presence of five features, with at least two being major diagnostic criteria)	Patient’s characteristics
Major Yamaguchi criteria:	
1. Fever of at least 39 °C lasting at least one week.	+
2. Arthralgias or arthritis lasting two weeks or longer.	-
3. Typical rash (maculopapular, nonpruritic) during febrile episodes.	-
4. Leukocytosis (10,000/µL or greater), with at least 80 percent granulocytes.	+
Minor Yamaguchi criteria:	
1. Sore throat	-
2. Lymphadenopathy	+
3. Hepatomegaly or splenomegaly	+
4. Abnormal liver function studies	-
5. Negative antinuclear antibodies and rheumatoid factor.	+
Exclusions	
1. Infection, especially sepsis and infectious mononucleosis	+
2. Malignancies, especially lymphomas	+
3. Rheumatic diseases known to mimic ASD	+

In conclusion, we describe a case of AOSD associated with formation of granulomatous lesions in lymph nodes, liver and presumably spleen. To the best of our knowledge, this is the first case of hepatic granulomas and the second case of lymph nodes’ suppurative necrotizing granulomas associated with AOSD. This case indicates that despite the rarity of granulomas formation in involved organs in AOSD, this finding should not deter the consideration of AOSD as a potential diagnosis in a compatible clinical context and after exclusion of other more common etiologies of tissue granulomas formation.
